# Resolutions of the Ohio College of Dental Surgery on the Death of Dr. George Watt

**Published:** 1893-04

**Authors:** 


### Resolutions of the Ohio College of Dental Surgery on the Death of Dr. George Watt.

The members of the Faculty of the Ohio College of Dental Surgery have learned with deep sorrow of the death of Dr. George Watt.

Dr. Watt had filled with unexampled ability the chair of Chemistry in this institution in its earlier years, and in that position received the well earned credit of being the distinguished pioneer of dental chemistry, linking thus in indissoluble bonds the youthful profession of his choice to that science, which above all others has given with unstinted hand the most generous and useful donations to the treasury of nascent dentistry.

It was not, however, in chemistry alone that Dr. Watt's eminence as a teacher was recognized. Those of us who attended his lectures in this college during the session of 1867–8 on pathology and therapeutics remember with admiration his lucid, sharply defined analysis of the abstruse questions belonging to these subjects. And while those who knew him could not but acknowledge his overmastering intellectuality, those who knew him best were those who loved him most.

In this spirit we lovingly offer this tribute to his memory and extend to his afflicted family our deepest sympathy.

